# A retrospective study of animal leptospirosis in Kazakhstan

**DOI:** 10.5455/javar.2024.k793

**Published:** 2024-06-12

**Authors:** Zhumagul Kirkimbayeva, Birzhan Biyashev, Svetlana Yermagambetova, Gulnur Kuzembekova, Bek Abdeliev

**Affiliations:** 1 Department of Microbiology, Virology and Immunology, Kazakh National Agrarian Research University, Almaty, Kazakhstan; 2 Reference Laboratory, National Scientific Center for Especially Dangerous Infections named after Masgut Aikimbaev, Almaty, Kazakhstan

**Keywords:** Serotype, antibody titer, molecular genetic diagnosis, focus of infection, monitoring

## Abstract

**Objective:**

The purpose of the paper was to monitor the disease incidence in farm and wild animals in some areas of Kazakhstan, which are most susceptible to leptospirosis, and the typification of isolated pathogens, carried out under the scientific and technical program “Studying the epizootological characteristics of the country territory on particularly dangerous diseases and developing veterinary and sanitary measures to improve their effectiveness” in 2021–2023.

**Materials and Methods:**

The material included the reports of veterinary laboratories on leptospirosis in recent years, as well as laboratory tests on samples carried out at the “SANA” research and development enterprise. During this period, 6,701 serum samples from farm animals and 86,651 serum samples from rodents were tested by enzyme-linked immunosorbent assay.

**Results:**

The serological results showed antibody titers in the blood of 6.32% of cattle, 5.4% of sheep, 4.2% of horses, and 1.8% of pigs. The highest number of positive samples were found in Turkestan (12.3%), Almaty (11.7%), and Kyzylorda (11.4%) regions. Infection in rodents was lower and ranged from 0.34% to 0.07% during these years. The population of leptospira-causing diseases of animals on the territory of the country is represented by 8 serogroups. Studies in 2022 on the detection of pathogenic leptospires by polymerase chain reaction in 350 samples of blood serum from animals and 350 samples of biomaterial from rodents from different regions of Kazakhstan were negative.

**Conclusion:**

Studies conducted as part of this work will help reduce the incidence of disease among the population and animals in Kazakhstan.

## Introduction

Leptospirosis is a dangerous infectious anthropozoonotic disease that is one of the most common diseases in the world. Outbreaks of leptospirosis are registered regularly in different countries on all continents. In their paper, Sykes et al. [1] point out that this disease is registered in more than one million people annually, with a fairly high mortality rate (more than 5%). However, given the similarity of clinical signs with colds in the first stage of the disease, as well as the nonspecific course and low sensitivity of diagnostic tests, most cases of leptospirosis in humans remain unreported, which reduces the preventive measures in such cases. Similar difficulties in the early diagnosis of leptospirosis are reported by Guernier et al. [2]. According to the same authors, the highest percentage of diseases occurs in Southeast Asia (Sri Lanka, India, the Philippines, Korea, and Tanzania), Oceania, the Caribbean, Latin America, and East Africa. Lau et al. [3] report that most cases occur in the summer months and are tied to natural reservoirs, which is due to the active development of the pathogen in the aquatic environment. However, the main role in the spread of leptospirosis belongs to domestic and wild animals. In their paper, Narkkul et al. [4] point out that the main source of the pathogen is rodents, in which the disease occurs in a chronic, asymptomatic form, and they can maintain a natural focus of the disease in nature for a long time.

In addition to tropical and subtropical countries, leptospirosis is also quite often registered in countries with temperate climates in the form of endemic diseases [5]. Kazakhstan is no exception. In the Republic, leptospirosis in animals and humans is most often observed in the East Kazakhstan Region. According to Tagaeva et al. [6], a significant spread of rodents in the vast territories of this area contributes to the maintenance of the natural foci of the disease and does not allow for the elimination of the source of the disease. Thanks to state support, a set of measures aimed at the prevention of especially dangerous diseases is carried out in the republic every year. So, KZT 7.2 billion was allocated from the budget in 2021 for the vaccination of animals, including those with leptospirosis, and the sum of KZT 12.8 billion was allocated for diagnostic studies [7]. No less important approach for the elimination of leptospirosis was to carry out deratization in the unfavorable territory. This approach contributed to the reduction of morbidity among the population of Kazakhstan.

According to Tagaeva et al. [6], there were no cases of leptospirosis in humans in the last few years, which allowed a reduction of the incidence rate from the epidemic situation in 1997–2001 to sporadic cases in 2005–2018. Unfortunately, cases of the disease periodically occur among agricultural livestock. According to the head of the Mazhilis committee on agrarian issues, there was a 5-fold increase in the number of animal cases of leptospirosis in Kazakhstan in 2020–2021 [8]. Little attention has been paid to the issue of controlling the spread of leptospirosis in Kazakhstan. Searching through scientific databases revealed only a few articles on leptospirosis. The main source of the pathogen was domestic animals and rodents (up to 16.1 and 14.7%, respectively), while environmental objects account for only about 10%. Bacteriological studies conducted in veterinary laboratories showed the presence of 5 types of leptospira in pigs [9]. In this regard, the purpose of this paper is to monitor the incidence of agricultural and wild animals in several regions of Kazakhstan, which are most susceptible to the occurrence of leptospirosis, as well as to conduct typing of isolated pathogens.

## Materials and Methods

### Ethical approval

Permission was obtained from the appropriate authority for publishing the data generated by the institution from 2021 to 2023.

### Study period

This article is based on a study of leptospirosis incidence in farm and wild animals across various regions of Kazakhstan, conducted as part of the Ministry of Agriculture of the Republic of Kazakhstan’s scientific and technical program from 2021 to 2023.

### Data sources

The study utilized multiple sources, including a 10-year dataset of laboratory reports from the Committee of Veterinary Control and Supervision of the Ministry of Agriculture of Kazakhstan, providing insights into leptospirosis incidence among farm animals in Kazakhstan. Additionally, laboratory examinations of blood samples were conducted on domestic animals and rodents in 13 regions.

### Methodology

The laboratory diagnosis of leptospirosis involved a comprehensive approach, including microbiological, immunological, and molecular-biological methods. Serological studies followed the guidelines of GOST 25386–91 [10], and the etiological structure of leptospirosis was assessed using the microscopic agglutination test (MAT) with 14 serogroups. The study was conducted at the “SANA” research and development enterprise.

### Serological testing

Antibody titers against leptospirosis were determined through enzyme-linked immunosorbent assay (ELISA) using Immunoglobulin G ELISA test kits by Bioassay Technology Laboratory. Thermo Scientific equipment was employed for ELISA testing.

### Rodent infestation

Biomaterial collection and laboratory studies of rodent infestation were conducted jointly with specialists from the M. Aikimbayev National Scientific Center for Dangerous Infections, Ministry of Health of the Republic of Kazakhstan, using the indirect hemagglutination test and antigen neutralization reaction.

### Molecular genetic analysis

Leptospires were isolated using a polymerase chain reaction (PCR) in real time with primers targeting the lipL32 gene. The amplisense leptospira-FL [11] reagent kit was utilized for nucleic acid isolation from biomaterials.

### Data analysis

The research results underwent statistical processing using the “Excel” analysis package within the “Microsoft Office” software suite.

## Results

**Figure 1.**
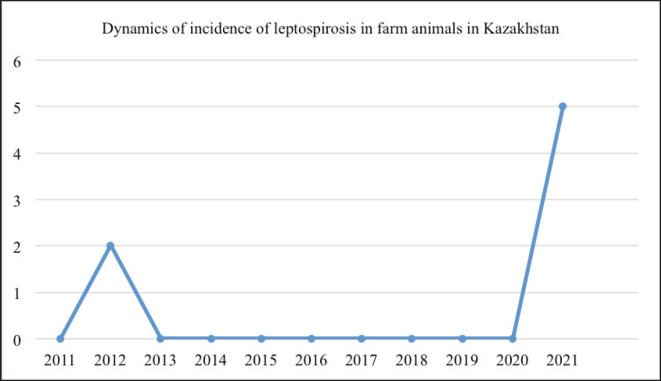
Incidence of leptospirosis in farm animals for the control period of 2011-2021.

An analysis of statistical data from laboratory tests and veterinary reports conducted by the Committee of Veterinary Control and Supervision of the Ministry of Agriculture of the Republic of Kazakhstan over the past decade (2012–2021) reveals that there were two officially reported cases of leptospirosis in farm animals during this period. The first case occurred in 2012, when foci of infection were established in two regions: Almaty and Zhambyl. The second one was established 8 years later, in 2021, when there was an increase in the incidence of the disease among farm animals in 5 regions: Almaty, Atyrau, Akmola, Karaganda, and Mangystau. At the same time, there were registered 2 cases of leptospirosis in cattle, 2 cases in horses, and 1 case in pigs. The dynamics of the epizootic situation in the country are shown in Figure 1.

In the period of 2020–2022, 6,701 blood serum samples from farm animals were tested in veterinary laboratories. Among the tests conducted, there were 3,465 samples from cattle, 2,887 samples from sheep, 189 samples from horses, and 160 samples from pigs. The test results are presented in Table 1.

**Figure 2.**
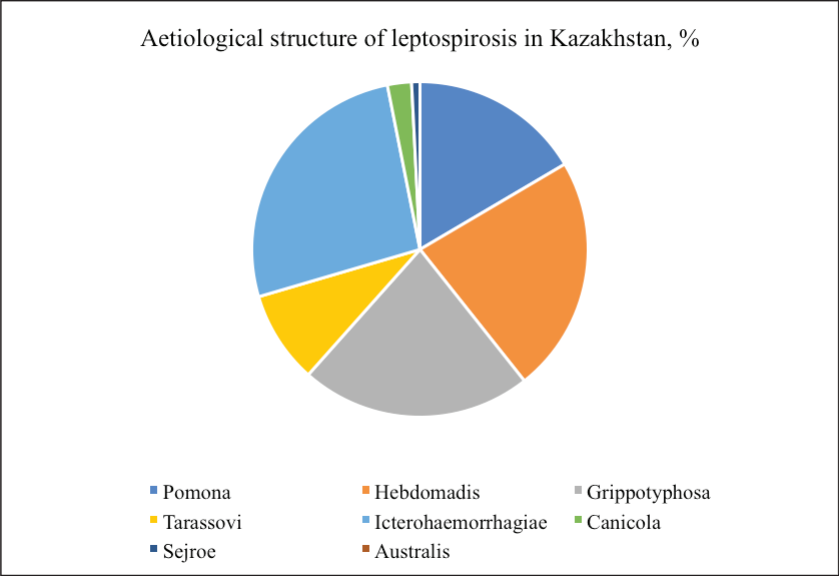
Ratio of leptospira serotypes isolated from farm animals in 2020-2022.

The results of the ELISA (enzyme-linked immunosorbent assay) demonstrated significant differences in the prevalence of seropositive individuals among farm animals in different regions of the country. Thus, among cattle (6.32%, *n =* 216), the largest number of positive samples was found in Turkestan (12.3%, *n =* 35), Almaty (11.7%, *n =* 23), and Kyzylorda (11.4%, *n =* 42) regions. A minimum number of cows positive for leptospirosis was found in Aktobe (1.9%, *n =* 8) and Akmola (1.6%, *n =* 9) regions. A similar picture was observed among other species of farm animals. Of the sheep examined, 5.4% (*n =* 155) were found to be infected with leptospira. Despite a smaller sample volume, positive animals were found in 4.2% of horses (*n =* 8) and 1.8% of pigs (*n =* 3). Leptospira serotyping from the control regions was performed by performing a microagglutination reaction. The results are presented in Figure 2.

The population of leptospira-causing diseases of farm animals in the country is represented by 8 serogroups: *Icterohaemorrhagiae, Hebdomadis, Grippotyphosa, Pomona, Tarassovi, Canocola, Sejroe, *and *Australis. *The *Hebdomadis *(33.3%), *Pomona *(19%), and *Icterohaemorrhagiae* (21%) serogroups prevail in the structure of leptospirosis etiology among cattle. Among infected sheep, leptospira serotypes *Icterohaemorrhagiae *(35.5%) and *Grippotyphosa *(30.3%) predominate. Antibodies to *Leptospira grippotyphosa *were detected in horses, and to *Leptospira pomona *in pigs*. *Single cases of detection of leptospira in the serogroups *Sejroe* and *Australis* can be regarded as nonspecific intergroup reactions, or these cases require further, more in-depth research on the serotyping of individual strains of leptospira. Leptospirosis is a focal infection, the spread of which is closely linked to rodents. Given the significant range of their distribution over vast areas of Kazakhstan, the elimination of this infection is quite problematic. Therefore, one of the methods of controlling the epizootic situation is continuous monitoring of the incidence of disease among rodents. For the control period of 2020–2022, serological studies of 86,651 blood serum samples of wild-caught rodents were conducted. The results obtained are shown in Table 2.

**Table 1.** Results of farm animal serum monitoring studies for detection of antibodies against leptospira by ELISA in 2020-2022.

Animal species	Number of tested samples	Number of positive samples	
		absolute value (n)	%
Cattle	3465	219	6.32
Sheep and goats	2887	155	5.4
Horses	189	8	4.2
Pigs	160	3	1.8
Total samples tested	6701	385	5.74

**Figure 3.**
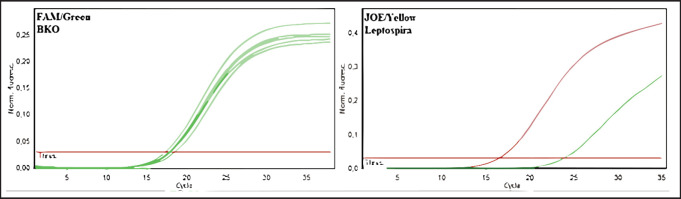
Amplification results of PCR products of biomaterial from rodents from Kyzylorda, Turkestan, East Kazakhstan, Atyrau, West Kazakhstan, Zhambyl, and Zhetysu regions (fluorescence curves and analysis parameters).

The greatest distribution of leptospira among the rodent population in 2020-2022 was observed in the Kyzylorda region—more than 16%—and this is given the relatively small number of surveyed rodents, whereas in other regions, the number of animals, in which blood antibodies were detected did not exceed 2%, or such animals were absent at all. Among rodents, leptospirosis infection was most common in animals of great gerbil, which prevailed among the examined species in the Zhambyl, Kyzylorda, and Aktobe regions. The monitoring research has shown negative dynamics in the revealing of seropositive animals for the last 3 years (from 0.34 to 0.07) that probably testify to a decrease in infectious processes in wildlife and the natural foci of disease. Results of deoxyribonucleic acids (DNA) testing of biomaterial samples from captured small mammals for detection of leptospira DNA can also lead to similar conclusions. For this purpose, pathological material from 2053 animals was analyzed. The results are summarized in Tables 3 and 4, as well as in Figure 3.

**Table 2.** Results of serological studies of rodent blood samples.

**Region**	2020		2021		2022 (8 months)		Entire period	
	Total samples examined, pcs	Positive samples, pcs	Total samples examined, pcs	Positive samples, pcs	Total samples examined, pcs	Positive samples, pcs	Total samples examined, pcs	Positive samples, pcs
Zhambyl oblast	1,589	48	2,199	18	718	4	4,506	70
Turkestan oblast and Shymkent city	341	0	475	0	259	3	1,075	3
Almaty region	2,016	0	5,064	0	5,869	4	12,949	4
Aktobe region	21,971	42	20,948	7	8,267	1	51,186	50
Kyzylorda region	105	16	260	44	14	2	379	62
Atyrau oblast	0	0	801	0	493	0	1,294	0
Mangystau oblast	2,827	0	4,591	0	2,767	0	10,185	0
Western Kazakhstan oblast	2051	0	2161	0	865	0	5077	0
Total	30,900	106 (0.34%)	36,499	69 (0.18%)	19252	14 (0.07%)	86,651	189 (0.22%)

**Table 3.** Results of PCR of samples of biomaterial from rodents from Kyzylorda, Turkestan, East Kazakhstan, Atyrau, West Kazakhstan, Zhambyl, and Zhetysu regions.

Samples	Sensitivity threshold value (Ct)	
	(JOE/Yellow) Leptospira	(FAM/Green) ВКО (STI)
Sample No. 1	23.92	17.42
Sample No. 2	neg.	neg.
Sample No. 3	neg.	neg.
Sample No. 4	neg.	neg.
Sample No. 5	neg.	neg.
Negative control	neg.	neg.
Positive control	16.49	-//-

**Table 4.** Summary results of quantitative PCR of biomaterial from rodents for DNA detection of *Leptospira *spp*.*

Region	2020-2022	
	Total samples examined, pcs	Of these, positive samples, pcs
Zhambyl	0	0
Turkestan and Shymkent city	182	1
Almaty	722	2
Aktobe	0	0
Kyzylorda	182	5
Atyrau	0	0
Mangystau	0	0
West Kazakhstan	967	0
TOTAL	2,053	8 (0.39%)

DNA of pathogens was isolated in the material from the Kyzylorda, Almaty, and Turkestan regions, i.e., in the most infected regions. More detailed studies on the incidence of the disease in animals in natural foci were conducted in 2022. Blood serum samples from 350 cattle and small cattle and 350 samples of biomaterial from rodents were examined (Table 5).

**Figure 4.**
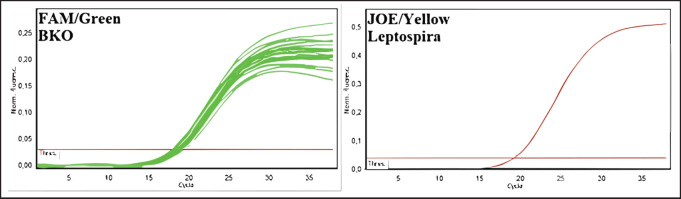
Amplification graphs of PCR products of 170 blood serum samples (34 pools) from cattle, sheep, and goats from Kyzylorda, Turkestan, and East Kazakhstan regions.

**Figure 5.**
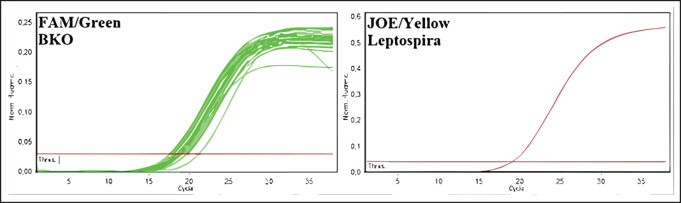
Amplification graphs of PCR products of 180 blood serum samples (34 pools) from cattle, sheep, and goats from Atyrau, West Kazakhstan, Zhambyl, and Zhetysu regions.

**Figure 6.**
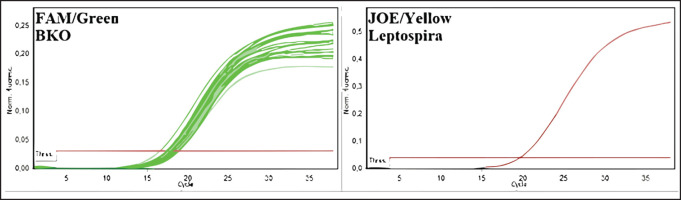
Amplification graphs of PCR products for 170 samples of biomaterial (34 pools) from rodents from Kyzylorda, Turkestan, and East Kazakhstan regions.

**Figure 7.**
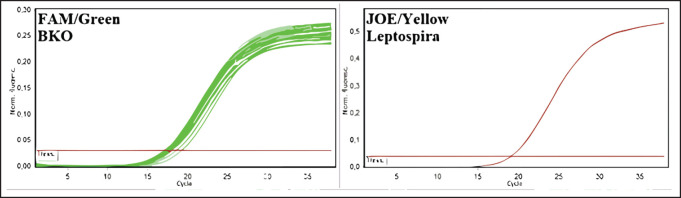
Amplification graphs of PCR products for 180 samples of biomaterial (34 pools) from rodents of Atyrau, West Kazakhstan, Zhambyl, and Zhetysu regions.

The selected samples were analyzed by the molecular genetic method using real-time polymerase chain reaction. In the initial phase of the research, 350 blood serum samples from cattle and sheep and 350 biomaterial samples from rodents were pooled into 68 pooled samples to expedite testing. Results were recorded by the fluorescence of amplification products. Negative results were stained green, and positive results were stained yellow. The test results and positive and negative controls are shown in Figures 4–7.

As a result of DNA testing of 350 samples of serum from farm animals and 350 samples of biomaterial from rodents in Kyzylorda, Turkestan, Eastern Kazakhstan, Atyrau, Western Kazakhstan, Zhambyl, and Zhetysu regions, specific sites of *Leptospira spp*. marker genes were not found.

## Discussion

The main method of diagnosing leptospirosis is a serological method called the MAT. According to Wilkinson et al. [12], the effectiveness of this method relies on understanding the local circulating serovars and conducting regular studies to maintain a comprehensive panel of antigens for accurate serological testing. Enzyme immunoassays and molecular methods are increasingly being used to diagnose leptospirosis more rapidly [13–15].

The monitoring study in Kazakhstan revealed sporadic cases of leptospirosis in farm animals, with pathogenic leptospira detection occurring at a relatively low frequency (<0.2%/year) among wildlife, a pattern similar to a study in the southern United States [16]. Leptospires consistently circulate among wild rodents, leading to ongoing environmental contamination with leptospira-infected urine, especially in freshwater bodies. However, leptospira do not reproduce in these bodies. Notably, in Kazakhstan, there were 8 years between two extreme cases of the disease in animals, with the second case affecting five regions, possibly due to rodent population spread and migration. Sporadic cases in animals tend to increase during seasonal rains, floods, or intense heat, leading to standing water bodies drying up and heightening the risk of disease [17]. While the specific trigger mechanisms for leptospirosis spread from reservoirs in Kazakhstan remain to be determined, it is evident that natural conditions play a significant role. The extended intervals between disease manifestations cannot be solely attributed to changes in rodent populations or their infection rates.

Diagnosing latent leptospirosis is challenging, typically relying on antibodies in unvaccinated animals’ blood [18]. Antibody titer determination is effective but often detects antibodies in outwardly healthy animals during monitoring studies, possibly due to atypical pathogen types [19]. Studies in different countries support this notion. Alashraf et al. [20] reported seropositivity in cats, dogs, and shelter workers in Malaysia without symptoms. This suggests that the true epizootic and epidemiological situation in Kazakhstan may differ from official records.

DNA testing tools like PCR are commonly used for diagnosing animal leptospirosis and detecting specific leptospira genome regions. Commercial tests often use primers to target the lipL32 gene, which encodes leptospira’s outer membrane protein and is more informative than other systems [21].

In this research, an AmpliSense Leptospira-FL real-time PCR system, based on lipL32 gene detection, was employed. Negative results in serum samples from farm animals and biomaterial from rodents caught in leptospirosis foci in 2022 suggest the absence of the disease among animals. However, detecting the latent chronic form of leptospirosis in animals remains challenging as it typically presents with mild or implicit symptoms, making diagnosis rare [18].

Real-time PCR studies may sometimes fail to detect leptospira genetic material in blood samples from animal foci [22]. This is because leptospira in most domestic animals are mainly found in the kidneys, their excretion into the environment is cyclic and not intense, and these animals often have low antibody titers, complicating both direct and indirect diagnosis [23]. Therefore, perhaps, the wrong material for monitoring studies of disease incidence in farm animals in Kazakhstan did not allow for the identification of pathogens in the genetic material from blood serum. Therefore, in the opinion of A.P. Loureiro and W. Lilenbaum [24], taking into account the predominant distribution of reproductive problems in the silent form of the disease in most species of farm animals, a possible material for the diagnosis of the chronic form of leptospirosis in addition to urine should be extractions from reproductive organs, which is especially appropriate in ruminants.

## Conclusion

Based on our leptospirosis monitoring studies in Kazakhstan among agricultural, domestic, and wild animals, the following key findings and recommendations emerge: Leptospirosis natural foci are prevalent in Kazakhstan, particularly in the southern regions. Small mammals had minimal contact with pathogenic leptospira (0.2% on average over 3 years), suggesting the presence of these foci. Serological studies indicate the circulation of pathogenic leptospires among farm animals, posing a direct threat to the population. Leptospirosis in farm animals in Kazakhstan involves eight serogroups. The predominant serogroups vary by livestock species. Seropositive wild rodents decreased from 0.34% to 0.07% over the past 3 years, possibly indicating a decline in the infection process. Several weather and other environmental factors should be analyzed, that could be a trigger for the development of leptospirosis outbreaks in 2012 and 2020.

**Table 5.** Geodata of blood sampling locations in Kazakhstan, summer–autumn of 2022.

No.	Sampling location (region, district, coordinates)	Number of blood sera from cows and sheep	Place of sampling from rodents (region, district, coordinates)	Quantity of biomaterials from rodents
1	Kyzylorda Region, Kosshynyrau rural district of Kyzylorda, N 44° 53’ 49.9488” E 65° 34’ 16.6656”	50/50	Kyzylorda Region, vicinity of Starorechye Zhanadarya FERs N 44º 18.259 E 64º 34.531, Ustyurt Ashikol N 45º 22.372 E 66º 13.254, Northeast Kyzylkum N 44º 17.365 E 65º 12.652,	50/50
2	Turkestan Region, Alfarabiya district of Shymkent city, Abaysky, Enbekshinsky, Ordabasinsky, Sayramsky, Tulkubassky, Kazygurtsky, Tolebiysky districts N 42º 25´ E 69º 38´, N 43º 02´ E 69º 53´, N 44º 08´ E 68º 11´, N 43º 09´ E 67º 51´,	50/50	Turkestan Region, Karatau, Baidibek, Sozak, Sauran districts N 42º 25´ E 69º 38´, N 43º 02´ E 69º 53´, N 44º 08´ E 68º 11´, N 43º 09´ E 67º 51´,	50/50
3	East Kazakhstan Region, Glubokovsky district, Bobrovka village, N 50º 14.077´ E 82º 71.041´	50/50	East Kazakhstan Region, Glubokovsky district, vicinity of the village of Glubokoe, N 50º 08.458´ E 82º 56.243´	50/50
4	West Kazakhstan Region, Baiterek, Chingirlau, Burlinskiy, Terektinskiy districts	50/50	Western Kazakhstan Region, vicinity of Podstepnovsky, Yanvartsevsky, Chebotarevsky, Rubizhensky, Chingirlau, Aksuatsky rural districts related to Terektinsky, Baytereksky, Chingirlau districts	50/50
5	Atyrau Region, Atyrau, N 47º 39,374´ E 52º 45,295´	50/50	Atyrau Region, Atyrau, Karabatan neighborhood, N 47º 17.586´ E 52º 21.068´	50/50
6	Jetisu Region, Yeskeldinskiy district, Karatal village, N 44º 88.051´ E 78º 72.034´, Jetisu Region, Yeskeldinskiy district, Karatal village, N 44º 88,040´ E 78º 71,086´,	50/50	Jetisu Region, Aksu district. Potentially focal area, N 45º 15.463´ E 79º 26.213´ Jetisu Region, Aksu district. West Jungar FER, N 45º 14.433´ E 79º 32.681´	50/50
7	Zhambyl Region, Moyinkum district, Kenes village N 43°57’25.05’’ E 73°31’17.86’’, Birlik N 44°3’14.39’’ E 73°36’38.01’’, Nazarbekova N 44°8’12.89’’ E 73°27’40.30’’, Zhaylymys N 44°08’12.46’’ E 73°34’35.41’’, Kokzhelek N 44°14’12.09’’ E 73°17’54.64’’, Kushaman N 44°14’39.90’’ E 73°13’50.08’’, Zhambyl N 44°18’19.36’’ E 73°6’57.10’’, Moyinkum N 44°17’49.34’’ E 72°56’13.28’’, block No. 27 N 44°13’45.64’’ E 73°04’52.37’’	50/50	Zhambyl Region, Moyinkum district, Sambet tracts vicinity N 43°48’34.15’’ E 73°28’05.57’’, Aktobe N 43°48’48.11’’ E 73°33’37.37’’, Alibek N 43°57’22.63’’ E 73°3’46.13’’, Ogisolgen N 44°07’29.64’’ E 73°12’48.37’’, Sazanbai N 43°57’35.64’’ E 73°19’43.37’’, Zhuangbai N 43°53’21.64’’ E 73°26’35.37’’, Estemes N 44°28’18.32’’ E 73°26’18.42’’, Orken N 43°04’58.63’’ E 73°04’58.13’’, Kainar N 43°53’45.05’’ E 73°34’54.86’’, Ashatay N 43°53’16.64’’ E 72°56’43.37’’, Dalankudyk N 43°42’11.64’’ E 73°03’45.37’’, Karakudyk N 43°42’07.64’’ E 73°20’24.37’’.	50/50
Total		350		350

## List of Abbreviations

ANR, antigen neutralization reaction; DNA, deoxyribonucleic acids; ELISA, enzyme-linked immunosorbent assay; IHT, indirect hemagglutination test; IgG, Immunoglobulin G; RT-PCR, real-time polymerase chain reaction.
